# Bio-Inspired Redox-Adhesive Polydopamine Matrix for Intact Bacteria Biohybrid Photoanodes

**DOI:** 10.1021/acsami.2c02410

**Published:** 2022-05-31

**Authors:** Gabriella Buscemi, Danilo Vona, Paolo Stufano, Rossella Labarile, Pinalysa Cosma, Angela Agostiano, Massimo Trotta, Gianluca M. Farinola, Matteo Grattieri

**Affiliations:** † Dipartimento di Chimica, Università degli Studi di Bari “Aldo Moro”, via E. Orabona 4, Bari 70125, Italy; ‡ IPCF-CNR Istituto per i Processi Chimico Fisici, Consiglio Nazionale delle Ricerche, via E. Orabona 4, Bari 70125, Italy; § CNR-NANOTEC, Institute of Nanotechnology, Consiglio Nazionale delle Ricerche, via E. Orabona 4, Bari 70125, Italy

**Keywords:** biophotoanode, photobioelectrochemistry, purple bacteria, quinone quantification in polydopamine, semiartificial photosynthesis, redox polymers

## Abstract

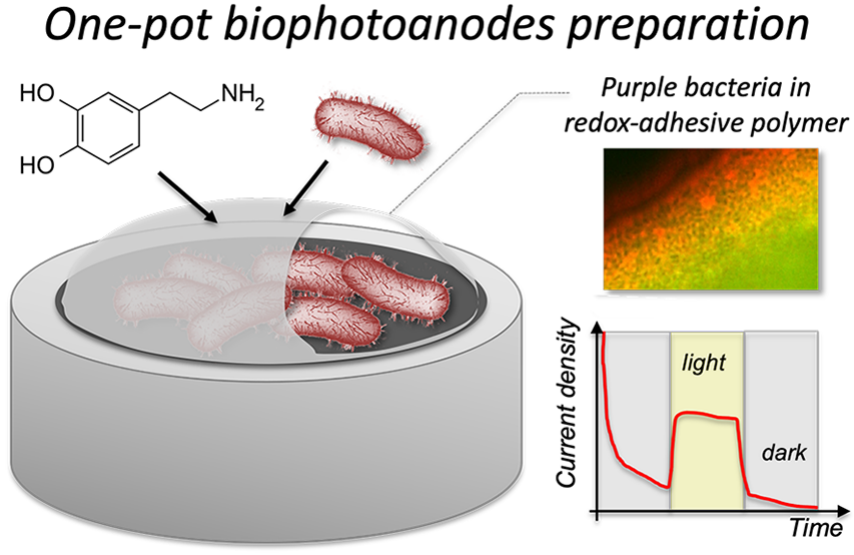

Interfacing intact and metabolically active photosynthetic bacteria with abiotic electrodes requires both establishing extracellular electron transfer and immobilizing the biocatalyst on electrode surfaces. Artificial approaches for photoinduced electron harvesting through redox polymers reported in literature require the separate synthesis of artificial polymeric matrices and their subsequent combination with bacterial cells, making the development of biophotoanodes complex and less sustainable. Herein, we report a one-pot biocompatible and sustainable approach, inspired by the byssus of mussels, that provides bacterial cells adhesion on multiple surfaces under wet conditions to obtain biohybrid photoanodes with facilitated photoinduced electron harvesting. Purple bacteria were utilized as a model organism, as they are of great interest for the development of photobioelectrochemical systems for H_2_ and NH_3_ synthesis, biosensing, and bioremediation purposes. The polydopamine matrix preparation strategy allowed the entrapment of active purple bacteria cells by initial oxygenic polymerization followed by electrochemical polymerization. Our results unveil that the deposition of bacterial cells with simultaneous polymerization of polydopamine on the electrode surface enables a 5-fold enhancement in extracellular electron transfer at the biotic/abiotic interface while maintaining the viability of the cells. The presented approach paves the way for a more sustainable development of biohybrid photoelectrodes.

## Introduction

Biohybrid electrochemical systems, where biological catalysts are coupled to abiotic electrodes, represent a sustainable approach for a variety of technological applications spanning from biosensing and water quality monitoring,^1−4^ bioelectrosynthesis,^5−9^ and micro to low power generation.^10−12^ Additionally, the use of photosynthetic entities as the biocatalyst allows utilizing sunlight, one of the most attractive energy sources, to power such systems, paving the way to the field of semiartificial photosynthesis.^13−17^ Using whole, metabolically active, microorganisms greatly simplifies the preparation of the biocatalyst (no enzyme isolation/purification required) and potentially enhances stability of the system thanks to their self-repairing and replication features. Purple nonsulfur bacteria have been used as model organisms for studying bacterial photosynthesis.^18,19^ Additionally, purple bacteria are of great interest for their potential application for H_2_ synthesis,^5,20^ as well as bioremediation and biosensing,^21^ with *Rhodobacter capsulatus* (*R. capsulatus*), representing a very interesting candidate as biophotocatalyst due to their extreme metabolic versatility.^22^ However, the cell membranes of purple bacteria, and microorganisms in general, act as insulating material hindering the transfer of electrons from their redox active sites to the electrode surface.^23^ Recent reports highlighted the photoinduced electron transfer process (both anodic and cathodic) in purple bacteria,^24,25^ and the approaches developed to accomplish and artificially tune the extracellular electron transfer (EET) with intact photosynthetic bacteria.^26^ These include utilizing artificial redox mediating systems based on Osmium,^27−29^ or quinone-based redox polymers,^30^ electrode surface engineering,^31^ and synthetic biology.^32−34^ Quinone-based artificial redox mediating approaches have been reported for a variety of photosynthetic organisms due to their biomimetic properties, as they compete with the natural electron carriers in the microorganisms for photoinduced electron extraction.^35^ At the same time, commercial polymers or bioinspired materials have been utilized to immobilize biocatalyst, either enzymes or whole bacteria,^36^ maximizing stability of the biohybrid system.^37^ However, the separate synthesis of redox polymers and immobilization matrices complicate the preparation of biohybrid electrochemical systems, limiting their utilization. Herein, we targeted both the redox mediation and immobilization challenges by employing dopamine (DA), a well-known catecholamine neurotransmitter, which provides a flexible surface coating in its polymerized form, polydopamine (PDA), to develop purple bacteria-based biohybrid photoanodes. The interest in utilizing this polymeric material is based on the simultaneous presence of catechol and amine groups, which mimic the adhesive mussel byssus that allows adhesion in underwater conditions and under shear stress, thus providing an interesting solution for the functionalization of biosurfaces used in water environments.^38^ On the basis of the polymerization conditions of dopamine (i.e., time, pH, temperature, ionic strength, medium), polymers with different characteristics can be obtained. For instance, PDA prepared exclusively by electrochemical means is insulating,^39^ a characteristic absent if obtained by oxygenic polymerization.^40^

Dopamine monomers have been recently utilized to modify electrode materials, such as carbon nanotubes providing adhesive properties for the deposition of thylakoids composites for biophotocurrent generation,^41^ and silk fibroin electrodes in microbial fuel cells.^42^ The entrapment of isolated photosynthetic reaction centers from purple bacteria in PDA films or nanoaggregates for biophotoanode preparation has also been reported,^40,43^ where diffusible exogenous redox mediators were required to obtain PDA–enzyme biohybrid systems for energy purposes. PDA has also been utilized to modify various electrode surfaces for application in microbial electrochemical systems, enhancing surface area and bacterial cell loading,^44^ decreasing startup times,^45^ and accelerating interfacial electron transfer.^46^ In addition, PDA improved single-cell electrical wiring for the model electroactive bacterium *Shewanella oneidensis* MR-1,^47,48^ and enhanced the adsorption of self-secreted flavins by *Shewanella xiamenensis*, resulting in improved extracellular electron transfer.^49^ Furthermore, the encapsulation of electroactive biofilms with PDA protected them against acid shocks, which could enhance their applicability in real environment.^50^ However, the capability of PDA to harvest photoinduced electrons from the photosynthetic apparatus of intact photosynthetic bacteria, which do not secrete redox active molecules, has not been elucidated to date.

Here, we show that a one-pot procedure initiated by oxygenic polymerization followed by electrochemical polymerization leads to a PDA-redox-mediating matrix enabling photocurrent production in whole bacteria-based biohybrid photoanodes. The applicability of the biohybrid system was validated utilizing glassy carbon electrodes and homemade, polyhydroxylkanoates-based, porous electrodes.

## Materials and Methods

### Chemicals

All chemicals were used as received without further purification. Ultrapure Milli-Q water (18 MΩ cm^–1^) was used for the preparation of all the solutions.

### Bacteria Growth

*R. capsulatus* strain DSMZ 152 was obtained from Deutsche Sammlung von Mikroorganismen and Zellkulturen GmbH (DSMZ) and grown in a liquid growth medium prepared as previously reported.^24^ The pH of the medium was adjusted to 6.8 using 5 M NaOH prior sterilization at 125 °C for 25 min (Systec VX-55). Trace elements, MgSO_4_, CaCl_2_, FeSO_4_, and biotin were added after sterilization, by filtration through a 0.20 μm filter (Puradisc 25). Bacterial cells were grown in sterile 50 mL bottles, sealed with airtight stoppers. An incandescent 80 W light bulb was used during the growth, performed at 28 °C in an incubator (IKA KS 3000 i control).

### Biohybrid Photoanodes Preparation

**Scheme 1:**
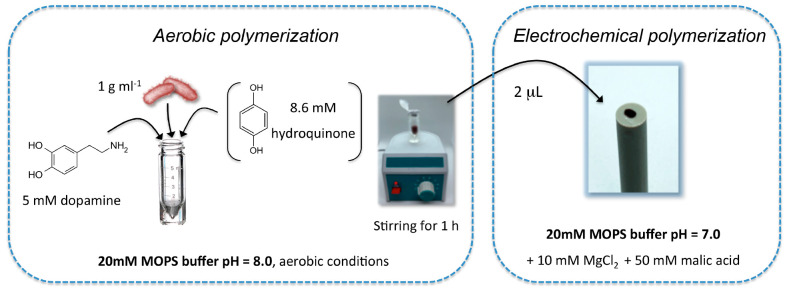
Biohybrid Photoanodes One-Pot Preparation with 60 min Aerobic Polymerization in MOPS Buffer at pH 8 Followed by the Electrochemical Polymerization by Repeated Cyclic Voltammetries in MOPS Buffer at pH 7 (20 Cycles at 20 mV s^–1^)

The procedure for the preparation of the biohybrid photoanode is reported in Scheme 1. Specifically, after 72 h growth, *R. capsulatus* cells were collected by centrifugation at 4000*g* for 20 min (Thermo Scientific Multifuge X3R). The cells were resuspended in 1 mL of 20 mM MOPS buffer (pH 8) + 10 mM MgCl_2_ + 50 mM malic acid and further concentrated by centrifugation at 12 000 rpm for 10 min (Giorgio Bormac S.r.l. Multispin 12). Finally, a solution with a bacterial cell concentration of 2 g mL^–1^ was prepared using 20 mM MOPS buffer (pH 8) + 10 mM MgCl_2_ + 50 mM malic acid. For the *R. capsulatus*–polydopamine biohybrid photoanode (PDA-*R*), an aliquot of the bacterial suspension was transferred to a 2 mL tube for a final concentration of the bacterial cells in the reaction tube of 1 g mL^–1^, together with 5 mM dopamine hydrochloride in MOPS buffer at pH 8.0. The solution was left to stir at 500 rpm (Metrohm 728 Magnetic Stirrer) for 1 h under aerobic conditions to start the polymerization of the dopamine monomer. This time was selected as PDA coating formation is highest in the early hours of polymerization and film formation properties are associated with early intermediates in dopamine autoxidation as recently reported.^51^ After this time, 2 μL of the obtained solution was dropcasted on a glassy carbon electrode (2 mm diameter) and left to dry for 20 min. The polymerization was then finalized electrochemically by repeated cyclic voltammetry at 20 mV s^–1^ (Autolab potentiostat PGSTAT302N). To obtain the *R. capsulatus*–polydopamine–hydroquinone biohybrid photoanode (PDA-HQ-*R*), 8.6 mM hydroquinone (HQ) was also added to the 2 mL tube before the aerobic polymerization step. Additionally, polyhydroxylkanoates (PHA)-based electrodes were prepared with commercially available vapor-grown carbon nanofiber (VG-CNF, 5 to 30% in weight) used as electrically conductive carbon filler, together with short chain length PHAs, namely poly-3-hydroxybutyrate (PHB) as biopolymer matrix. The 1 cm^2^ electrodes were cut, and 50 μL of the PDA-*R* solution was dropcasted and left to dry for 60 min prior to performance of the electrochemical polymerization step to obtain biophotoanodes on this support (PHB-PDA-*R*). It should be noted that the desiccation times utilized (20 and 60 min for glassy carbon and PHB-based electrodes, respectively) are consistently shorter than the 6–7 h desiccation times that were previously reported to affect biophotocurrent generation in *R. capsulatus*-based electrodes.^52^ Accordingly, the selected desiccation times are not expected to significantly affect the performance of the biohybrid electrodes.

### Epifluorescence Microscopy Studies

The viability and the effective entrapment of *R. capsulatus* cells into the PDA matrix were initially studied by means of epifluorescence microscopy. Specifically, after the 1 h aerobic polymerization with PDA-*R* in the 2 mL tube, the biohybrid matrix obtained was collected by centrifugation at 12 000 rpm and resuspended in the MOPS buffer pH 7.0. Following, 6 μL of fluorescein diacetate (FDA) (1 mg mL^–1^ in acetone) was added to 600 μL of the PDA-*R* suspension and incubated for 2 h. FDA is colorless and nonpolar. Intracellularly, or next to membrane-associated esterases produced by metabolically active microorganisms, FDA is hydrolyzed to a green fluorescent compound, fluorescein, which can be detected spectroscopically by measuring its fluorescence. Since dead cells cannot accumulate, or hydrolyze FDA, live/dead cells can be distinguished. Control experiments were performed following the same procedure but incubating 6 μL of FDA (1 mg mL^–1^) with 600 μL of *R. capsulatus* cells suspension only. Finally, the presence of the PDA layer surrounding the viable *R. capsulatus* cells was studied by adding 10 μL of rhodamine-G (0.5 mg mL^–1^ in water) to the PDA-*R* and *R. capsulatus*-only samples after the 2 h incubation with FDA. Rhodamine-G is a fluorescent stain capable of binding to PDA, confirming the presence of the polymer surrounding bacterial cells.

### Spectrofluorimetric Studies

The viability of *R. capsulatus* cells after entrapment in the PDA matrix was quantitatively determined by performing quintuplicates spectrofluorimetric measurements using living cells of *R. capsulatus*, living cells coated with PDA (PDA-*R*), dead cells obtained after heat-treatment for 4 h at 120 °C entrapped in PDA (PDA-HT_*R*), and lone PDA to demonstrate that only viable cells can convert diacetate residue into a fluorescent one. The various samples, prepared as previously specified, were collected by centrifugation at 7000*g* and resuspended in MOPS buffer at pH 7.0. Following, FDA (1 mg mL^–1^ in acetone) was added to all the suspensions and incubated for 2 h. Signals of fluorescent molecules were detected using a spectrofluorimetric approach (Varian Cary Eclipse Fluorescence Spectrophotometer), 488 nm excitation, 495–650 nm emission window, excitation slit 2.5, emission slit 5, 1 cm cuvette thickness.

### Electrochemical Setup

All of the electrochemical measurements were performed in triplicate, at 24 ± 1 °C in 20 mM MOPS buffer (pH 7) + 10 mM MgCl_2_ + 50 mM malic acid with the electrolyte exposed to air (aerobic conditions). Average values are reported together with one standard deviation. Photobioelectrocatalysis was investigated in a three-electrode electrochemical cell by cyclic voltammetry (CV) and amperometric *i*–*t* tests (Autolab potentiostat PGSTAT302N). The working electrode was the biohybrid photoanode prepared as previously described. Control experiments were performed with bare glassy carbon electrodes in the absence of *R. capsulatus* cells (sterile electrodes), as well as glassy carbon electrodes with only PDA or PDA-HQ. Furthermore, *R. capsulatus* cells were also heat-treated at 120 °C and utilized in the PDA matrix to confirm the biotic origin of the photocurrent. The counter electrode was a Pt foil, and the reference electrode was a Ag|AgCl (3 M NaCl, Basi MF2052) electrode. All potentials in this work refer to this reference electrode. For the controls with the polyhydroxylkanoates-based electrodes, the same procedure was followed, substituting only the substrate electrode. Illumination during the photoelectrochemical studies was provided with a fiber optic lamp (Schott KL 1500 LCD), equipped with a light bulb of 10 W. For the comparison of the cyclic voltammetries obtained with the different electrode configurations, current densities were evaluated considering the anodic scan obtained for the third cycle, at a potential of +0.32 V. For the comparison of the amperometric *i*–*t* traces obtained with the different electrode configurations on glassy carbon, the current densities were evaluated at the end of the third illumination cycle (25 min), and the biophotocurrents were calculated based on the average current densities under illumination (25 min) minus average current densities under dark after the third illumination cycle (28 min). Similarly, the photocurrents for the control, sterile, electrodes were calculated based on the current densities obtained at 25 and 28 min of the amperometric *i*–*t* test. For the PHB-based electrodes, (bio)photocurrent densities were calculated based on the average current densities under illumination (10 min) minus average current densities under dark (13 min). In this case, the first illumination cycle was utilized for the evaluation due to the drift in current response obtained for PHB-PDA-*R* electrodes over time.

### Hydroquinone Quantification in Polydopamine Matrix by Absorption Spectroscopy

The HQ entrapment into PDA structure was estimated by first derivate absorption spectroscopy of the supernatant obtained after oxygenic polymerization of DA. Specifically, increasing concentrations of HQ in MOPS buffer were initially prepared in the presence of 5 mM DA and analyzed by absorption spectroscopy. The selected final concentrations of HQ in solution were the following: 0, 0.5, 1, 1.6, 2.3, 3, 3.6, 4.3, and 4.9 mM. After UV–vis absorption measurements, the first derivate was applied to obtain a calibration curve using the characteristic peak at 302 nm. To ensure that DA/PDA do not share the selected peak at 302 nm, absorption measurements were performed for solutions containing both DA monomer and PDA resulting after 1 h of aerobic polymerization. The samples for HQ determination in PDA after oxygenic DA polymerization in the presence of 8.6 mM HQ where prepared by centrifugation at 7000*g* for 10 min. The obtained supernatant was immediately analyzed by absorption spectroscopy, determining the amount of HQ that was not entrapped in the PDA matrix. To further confirm the encapsulation of HQ into PDA matrix, a similar quinone (2,3-dichloro-5,6-dicyano-1,4-benzoquinone, DDQ) was used as probe due to the presence of nitrile groups that can be distinguished from DA/PDA moieties. DDQ was first solubilized in DMSO, and then added to a 5 mM of DA in MOPS buffer pH 8 with a final concentration of 4 mM. The solution was kept overnight under mild stirring, and exposed to air to promote oxidative polymerization of DA. The prolonged reaction time was due to the presence of DMSO (essential for DDQ solubilization), which resulted in a slower reaction rate. After polymerization, the solution was centrifuged at 7000*g* for 10 min. The supernatant was removed, and the PDA-DDQ pellet was washed two times with MOPS buffer at pH 7. The obtained polymers, namely PDA (in MOPS), PDA-DDQ (in MOPS), and DDQ (in DMSO/MOPS), were spotted onto glass and left to dry prior to be analyzed by FT-IR/ATR.

### Scanning Electron Microscopy

Electrodes morphology was observed by a MERLIN Zeiss field emission gun scanning electron microscope (FE-SEM) with an operating voltage of 5 kV, cutting samples of the desired size directly from the free-standing electrode and performing imaging analyses on such samples without any further manipulation.

## Results

**Figure 1:**
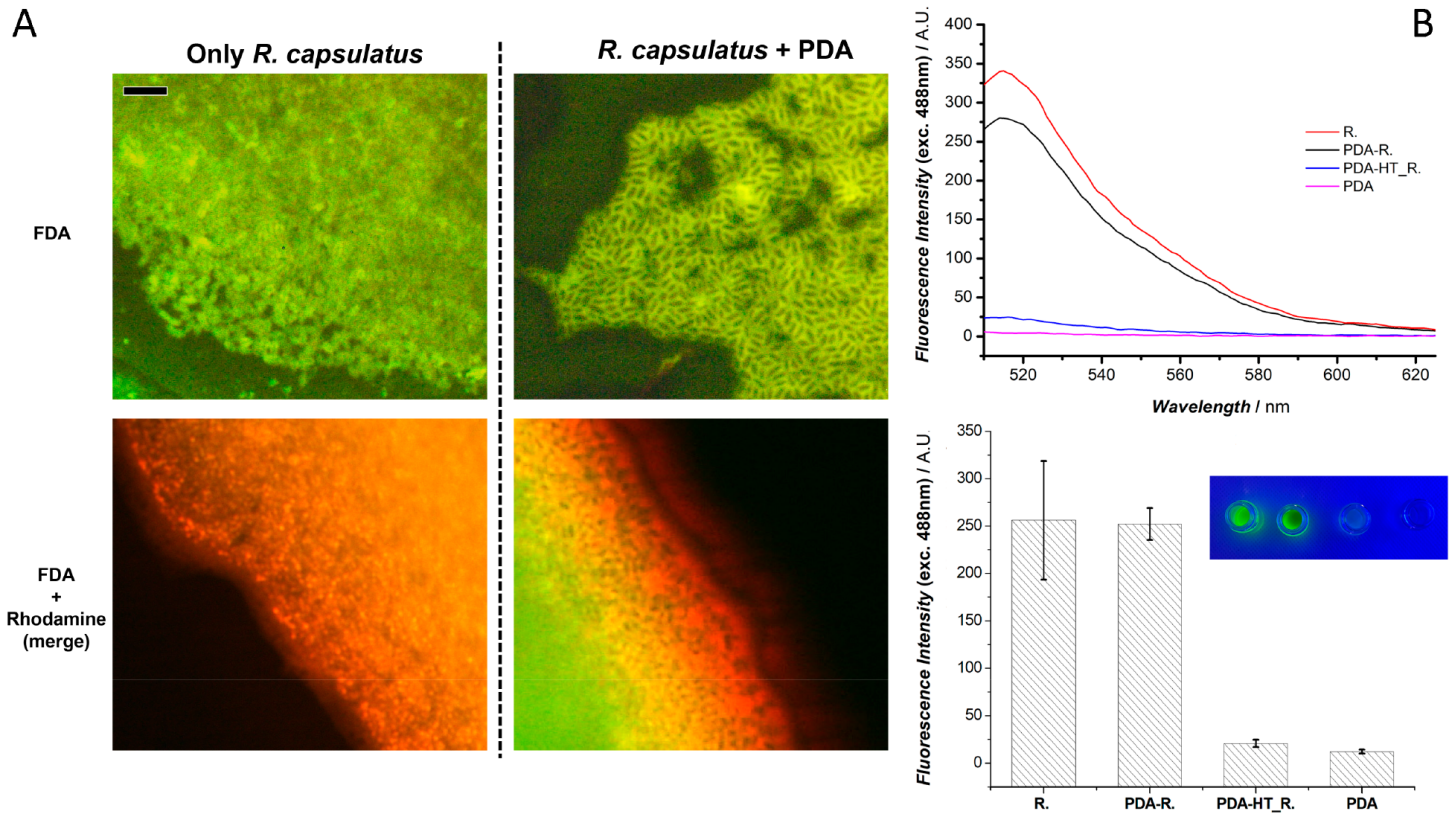
(A) Top (FITC filter cube set): Fluorescence microscopy images of *R. capsulatus* cells (left) and PDA-*R* matrix after a 2 h incubation with FDA. Bottom (combination merge of TRITC and FITC filter cube sets): Fluorescence microscopy images of *R. capsulatus* cells (left) and PDA-*R* matrix after a 2 h incubation with FDA plus overnight incubation with Rhodamine-G. Scale bar 10 μm. (B) Top: Representative fluorescence spectra of the different samples investigated; 488 nm excitation, 495–650 nm emission window, excitation slit 2.5, emission slit 5, 1 cm cuvette thickness. Bottom: Comparison of fluorescence intensities at 514 nm for the different samples, the inset shows an optical image of the four samples under blue light.

Among the possible strategies for tuning photoinduced extracellular electron transfer, the immobilization of the active components in redox matrices has been reported including Osmium^27−29,53^ and naphthoquinone-based redox polymers.^30^ However, these polymers require a separated synthetic procedure and lack of adhesive properties. For this purpose, dopamine was selected due to its capability to polymerize directly in a bacterium compatible environment while providing a multipurpose material with adhesion features and tunable conductive properties. Dopamine polymerization mechanisms have been extensively discussed in recent works, with various parallel polymerizations including covalent reactions and supramolecular interactions (hydrogen bond, cation−π, and π–π stacking), which lead to a PDA chemical structure being very complex and randomized.^54^ Here, we first investigated if *R. capsulatus* cells could tolerate exposure to dopamine monomer and its polymerization. Accordingly, FDA was added to the PDA-*R* suspension, and epifluorescence microscopy analysis of the obtained suspension was performed after a 2 h incubation. The obtained epifluorescence microscopy images are reported in Figure 1A showing that fluorescein diacetate is hydrolyzed to fluorescein, confirming the viability of the bacterial cells exposed to the dopamine monomer and coated with PDA (top right), as obtained for the control performed with free *R. capsulatus* cells (top left). Interestingly, a different compartmentalization of fluorescein is obtained for bare bacteria with respect to the polydopamine-coated cells, which could be due to the PDA matrix retaining the FDA molecule. Furthermore, to confirm that the PDA matrix surrounded the viable bacterial cells, rhodamine was also added together with fluorescein diacetate during dopamine polymerization in the presence of *R. capsulatus* cells. The rhodamine dye modifies the free polydopamine surface via noncovalent interactions (π–π stacking and hydrogen bonding), and its positive charge also promotes electrostatic interaction with the negatively charged polydopamine surface.^55^ The investigation via bidimensional fluorescence microscopy revealed a localized rhodamine fluorescence surrounding bacterial cells (bottom right), indicating that the polymer ubiquitously covered the viable cells as colocalized signals with FDA (yellow). Conversely, exposing *R. capsulatus* cells to rhodamine in the absence of dopamine resulted in a spread fluorescence of rhodamine with hidden or absent fluorescein emission and a nontypical round shape of the bacterial cells, indicating that rhodamine had a negative effect on cell viability. The results indicate that PDA not only provides a matrix for immobilization of viable bacterial cells but also has a protective role against inhibitor of bacterial cells activity, further supporting the use of this polymer for bacteria entrapment. Furthermore, the quantitative determination of *R. capsulatus* cells viability after entrapment in the PDA matrix was studied by spectrofluorimetric measurements (Figure 1B). As shown in the representative spectra (top) and by the intensity of emission histograms (bottom), we obtained a clear emission signal only with living *R. capsulatus* cells and with cells entrapped in PDA (PDA-*R*). The lone PDA polymer and heat-treated cells entrapped in PDA did not hydrolyze the diacetate moiety, clearly confirming that viable cells are present in PDA-*R*. ANOVA online statistical correlation has been applied to the histograms, and validation passes for *p*-value < 0.05.

**Figure 2:**
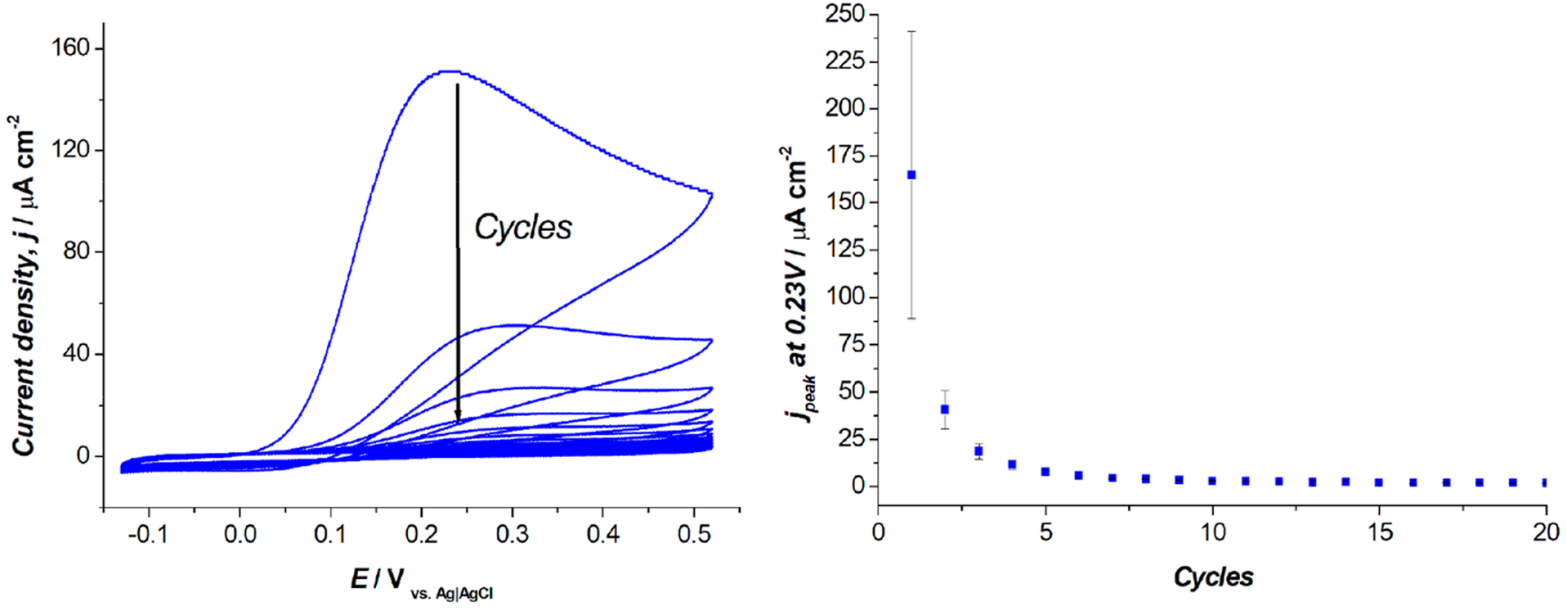
Left: Electropolymerization of the PDA-*R* matrix by repeated cyclic voltammetry. Scan rate, 20 mV s^–1^; CE, Pt; RE, Ag|AgCl 3 M NaCl. Right: Peak current density at +0.23 V versus cycle number.

Once the viability of the cells after 1 h aerobic PDA polymerization at pH 8 was confirmed, the obtained suspension was dropcasted on glassy carbon electrodes to perform the electrochemical polymerization step. DA oxygen-driven polymerization is rather slow,^51^ with a possible detrimental effect on cell viability. Conversely, pure electrochemical polymerization is considerably faster but leads to a dense and continuous PDA coating showing insulating properties,^39,56^ which might hinder its application in biophotoanodes. Here, after the aerobic polymerization, a series of 20 cyclic voltammetries at 20 mV s^–1^ was performed using an electrolyte at pH 7 (where the aerobic polymerization is extremely slow) to finalize the biohybrid photoanode (Figure 2, left). Over the CV cycles during the electrochemical reactions, the intensity of the dopamine oxidative peak at 0.23 V progressively decreased (Figure 2, right) and the PDA-*R* structure stabilized.

**Figure 3:**
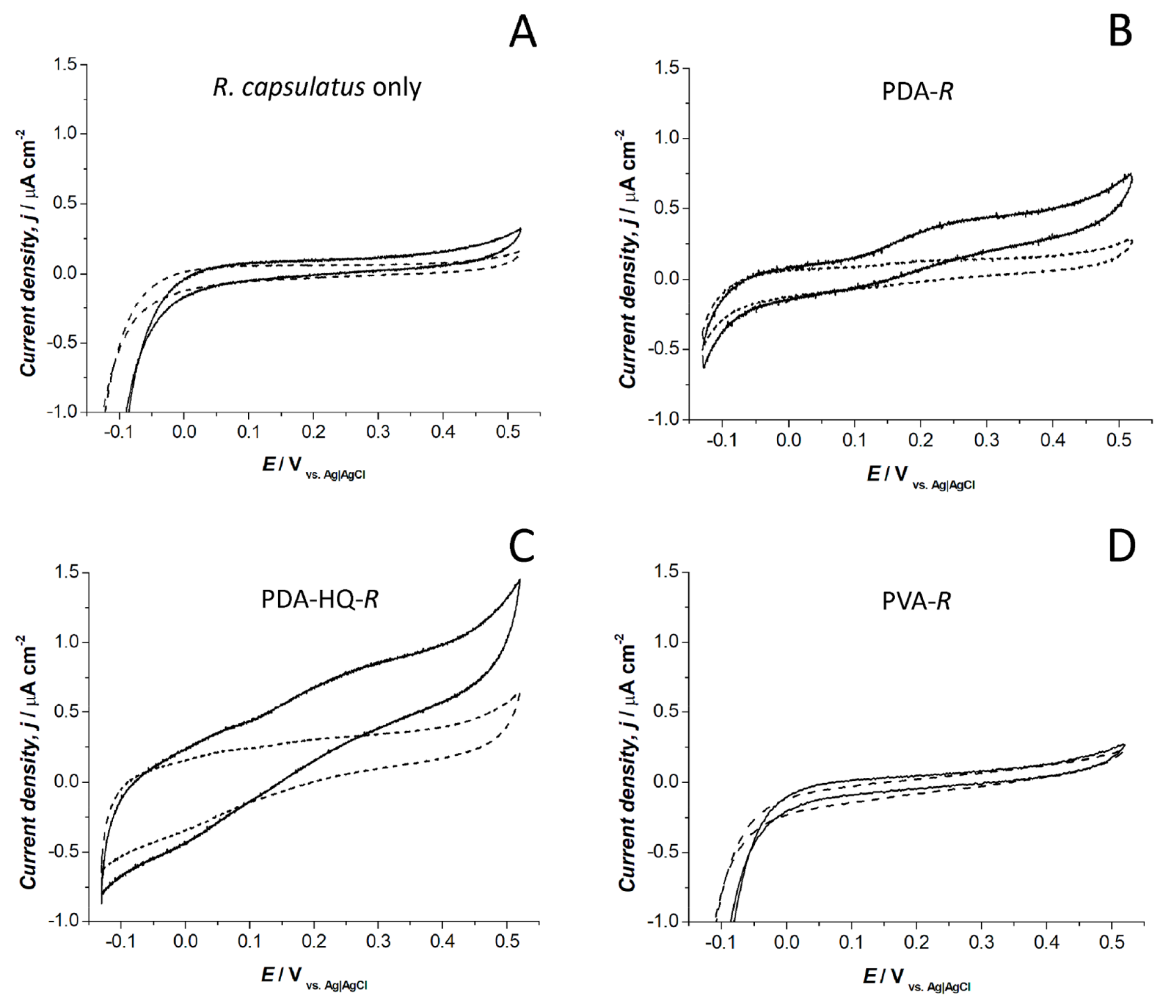
Cyclic voltammetry for the biohybrid photoanodes under light (continuous lines) and dark conditions (dashed lines). Scan rate, 1 mV s^–1^; CE, Pt; RE, Ag|AgCl 3 M NaCl. (A) *R. capsulatus* only; (B) PDA-*R*; (C) PDA-HQ-*R*; (D) PVA-*R*.

Photobioelectrocatalysis for the obtained biohybrid photoanode was then studied by performing 1 mV s^–1^ cyclic voltammetry, reported in Figure 3. A potential of +0.32 V was selected for comparing the photobioelectrocatalytic performance of the various electrodes as under those conditions a quasi-steady state current was reached for all the systems investigated. The biohybrid electrodes with bacteria deposited directly onto the electrode surface (Figure 3A) gave a minimal light response, with a current of 0.13 ± 0.01 and 0.11 ± 0.02 μA cm^–2^ at +0.32 V in light and dark conditions, respectively. Conversely, the PDA-*R* biohybrid systems revealed a clear light response, with an onset of the anodic current at 120 ± 20 mV, and current densities of 0.42 ± 0.03 and 0.16 ± 0.02 μA cm^–2^ at +0.32 V were recorded in light and dark conditions, respectively (Figure 3B). Furthermore, photobioelectrocatalysis for the biohybrid system obtained after adding 8.6 mM hydroquinone during dopamine polymerization revealed a comparable onset of the anodic current at 140 ± 10 mV (Figure 3C), and higher current in light and dark conditions (0.94 ± 0.05 and 0.30 ± 0.10 μA cm^–2^ at +0.32 V, respectively). It should be noted that while the redox peaks due to HQ entrapped in the PDA matrix are not clearly visible in the PDA-HQ-*R* biophotoanode, they can be identified from the cyclic voltammetry of the control electrode PDA-HQ (in absence of *R. capsulatus* cells) reported in Figure S1B (Supporting Information), with the pair of redox peaks having a formal redox potential of approximately +0.1 V versus Ag|AgCl (3 M NaCl) in accordance to previous literature.^24^ The biophotocurrent results obtained for the PDA-*R* biohybrid system can be explained thanks to the redox mediation properties of the prepared PDA matrix, where the available quinones shuttle the photoinduced electrons. In addition, PDA ensures bacteria cells adhesion at the biotic/abiotic interface, resulting in an improved contact between bacteria and electrode surface. Such conditions could enhance the role of endogenous quinones produced by the bacterial cells on current generation by shuttling the electrons from the photosynthetic apparatus to the electrode surface that is maintained in close proximity. Despite their origin being either endogenous or from the PDA matrix, quinones play a central role in the redox mediation process, as confirmed by the comparable onset of the anodic current obtained with PDA-HQ-*R* and the system composed of only PDA-*R*. Future studies should be focused on unveiling the detailed electron transfer process inside the PDA matrix. Here, the higher current reached for the PDA-HQ-*R* system compared to the PDA-*R* system can be explained with a higher abiotic photoresponse of the PDA-HQ system compared to the abiotic PDA system, as shown by control cyclic voltammetric studies (Supporting Information, Figure S1). Finally, the redox mediation role of the PDA matrix was further confirmed by comparing the PDA-*R* biohybrid system with a control biohybrid system obtained combining *R. capsulatus* cells with polyvinyl-alcohol (PVA), a known adhesive, optically transparent polymer having no redox mediation capability. The cyclic voltammetry under light/dark conditions for the control PVA-*R* photoanodes resulted in no photocurrent generation, as shown in Figure 3D.

**Figure 4:**
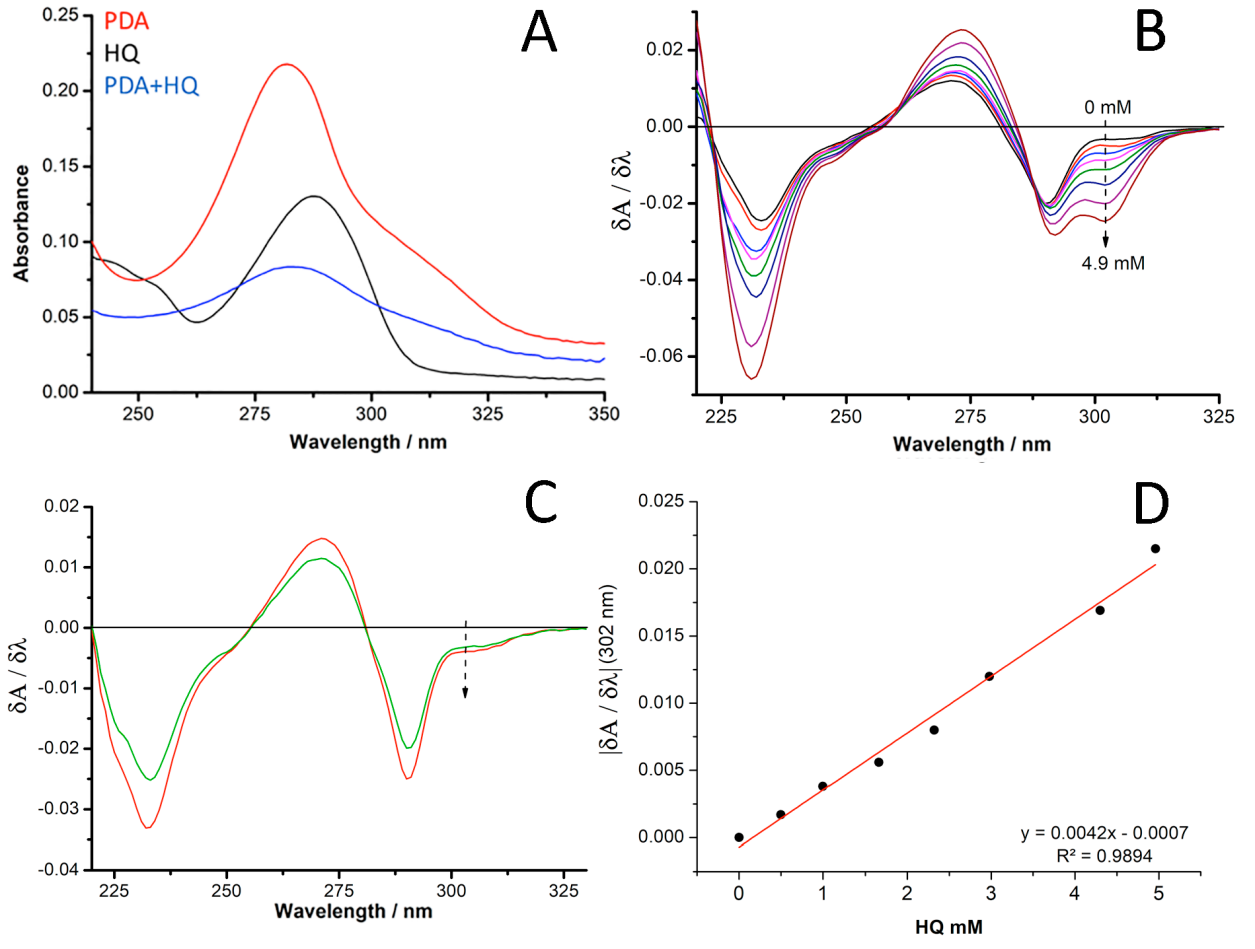
UV–vis absorption spectra of (A) PDA, HQ, and a mixture of PDA and HQ; (B) first derivate of absorption spectra of DA 5 mM with increasing concentrations of HQ indicated by the black arrow (0, 0.5, 1, 1.6, 2.3, 3, 3.6, 4.3, 4.9 mM); (C) DA at time 0 and after 1 h of aerobic polymerization; (D) calibration plot for HQ concentration versus first derivate of the absorption at 302 nm.

The entrapment of HQ in the control PDA-HQ-*R* matrix was confirmed by absorption spectroscopy of the supernatant obtained after the preparation of the matrix. It should be noted that the detection of HQ in the supernatant turned out to be more complicated than expected due to the fact that DA/PDA and HQ present similar functional groups, thus having overlapping absorption bands (Figure 4A). However, the first derivative of the absorption spectra allowed resolving of the contributions of DA/PDA and HQ. A calibration of HQ (both with and without DA) as absorption first derivate was performed: by increasing HQ concentrations, a peak at 302 nm was obtained, and increases for increasing concentrations of HQ (Figure 4B). The same peak is not present for PDA alone (characterized by the peak at 290 nm, Figure 4C), thus allowing separation of the absorption response of HQ while in the presence of DA. On the basis of the first derivative of the UV–vis spectra obtained for the supernatant of PDA-HQ-*R* preparation, a calibration curve was obtained (Figure 4D). The concentration of HQ in the supernatant after oxygenic polymerization was 6.5 ± 0.3 mM; thereby, the average concentration of HQ in the PDA-HQ-*R* biohybrid matrix was 2.1 ± 0.6 mM. Furthermore, the possibility to coimmobilize quinone mediators in the PDA matrix was investigated by FT-IR/ATR directly using the PDA matrix with DDQ as probe (Figure S2, Supporting Information). The detection of a peak at 2211 cm^–1^ related to the stretching of triple bond in C≡N for the PDA modified with DDQ confirmed the effective coimmobilization of the mediator into PDA matrix. This characteristic signal of DDQ is not present in the bare PDA control (blue line), providing additional evidence of the successful entrapping of quinones in the PDA matrix.

**Figure 5:**
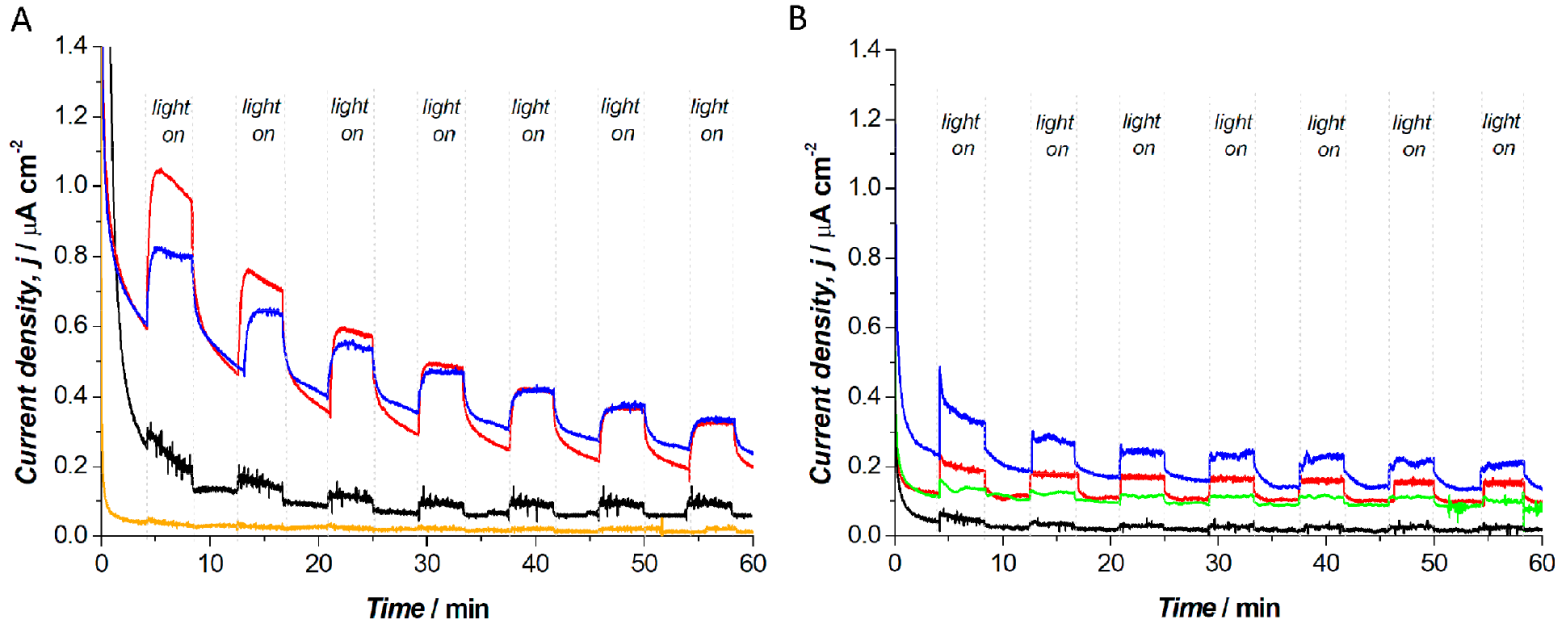
Amperometric *i*–*t* traces at +0.32 V versus Ag|AgCl 3 M NaCl under light/dark conditions for: (A) biohybrid photoanodes (red, PDA-*R*; blue, PDA-HQ-*R*; black, *R. capsulatus* only; orange, PVA-*R*); (B) control electrodes (red dots, only PDA; blue dots, only PDA-HQ; black, bare glassy carbon electrode; green, dead *R. capsulatus* cells in PDA matrix).

Photobioelectrocatalysis was further studied by chronoamperometry performed at +0.32 V versus Ag|AgCl 3 M NaCl for all the biohybrid photoanodes prepared (Figure 5A). This potential was selected based on the cyclic voltammetry results, which revealed that under this condition a quasi-steady state current is obtained. Furthermore, for the best comparison of the current densities obtained, the first two light cycles were not considered, and only the response at the third illumination cycle is discussed (obtained after 25 min of polarization at +0.32 V). This allowed the stabilization of the current response obtained with all the abiotic controls (Figure 5B) and their optimal comparison with the biotic systems. The system with PDA-*R* showed the highest current response (red line), reaching 0.44 ± 0.08 μA cm^–2^ at the third illumination cycle, for a biophotocurrent of 0.18 ± 0.08 μA cm^–2^. Only the system having the modified PDA-HQ-*R* biohybrid photoanodes showed comparable performance (blue line), reaching 0.52 ± 0.02 μA cm^–2^ at the third illumination cycle, for a biophotocurrent of 0.17 ± 0.03 μA cm^–2^. The biophotoanodes prepared with only *R. capsulatus* cells on the glassy carbon electrodes showed a limited biophotocurrent due to the hindered direct photoinduced electron transfer between bacteria and the electrode surface (black, 0.03 ± 0.01 μA cm^–2^). Entrapping *R. capsulatus* cells in the PVA matrix resulted in no photoresponse (orange), confirming the results previously discussed for the cyclic voltammetry studies. Various control electrodes were prepared to confirm both the biotic origin of the photocurrent and the role of the PDA matrix for photoinduced electron transfer (Figure 5B). Specifically, utilizing heat-treated bacterial cells in the PDA matrix, no significant photoresponse was obtained (green dots), with light/dark currents comparable to a bare glassy carbon electrode (black dots). The control electrodes prepared with abiotic PDA (red dots) and PDA-HQ (blue dots) matrices gave a limited photoresponse, reaching 0.05 ± 0.04, and 0.08 ± 0.03 μA cm^–2^, respectively. It is interesting to note that the abiotic PDA-HQ system gave a slightly higher photoresponse compared to the abiotic PDA system despite the latter performing better in the biohybrid system, thus highlighting the biophotocurrent production in the PDA-*R*. Furthermore, when comparing the cyclic voltammetry results of Figure 3 and the amperometric *i*–*t* traces of Figure 5, it could be noted that PDA-HQ-*R* gave the highest current density under illumination in the cyclic voltammetry, while comparable current densities are obtained for PDA-HQ-*R* and PDA-*R* in the amperometric studies. This result can be explained considering the higher capacitive contribution to the current response in the case of the PDA-HQ-*R* biophotoanode, as shown by the cyclic voltammetry studies. However, in amperometric *i*–*t* studies, the capacitive component of the current normally falls to zero in short times (up to a few seconds depending on the system under investigation), thus allowing comparison of the current resulting from only faradic processes in both PDA-HQ-*R* and PDA-*R* biophotoanodes. Finally, it should also be noted that even after the 1 h amperometric *i*–*t* characterization, no HQ could be detected in the spent electrolyte, as shown in Figure S3, indicating that the PDA matrix can successfully entrap the HQ and the evolution of the photoresponse is not due to loss of HQ in solution.

**Figure 6:**
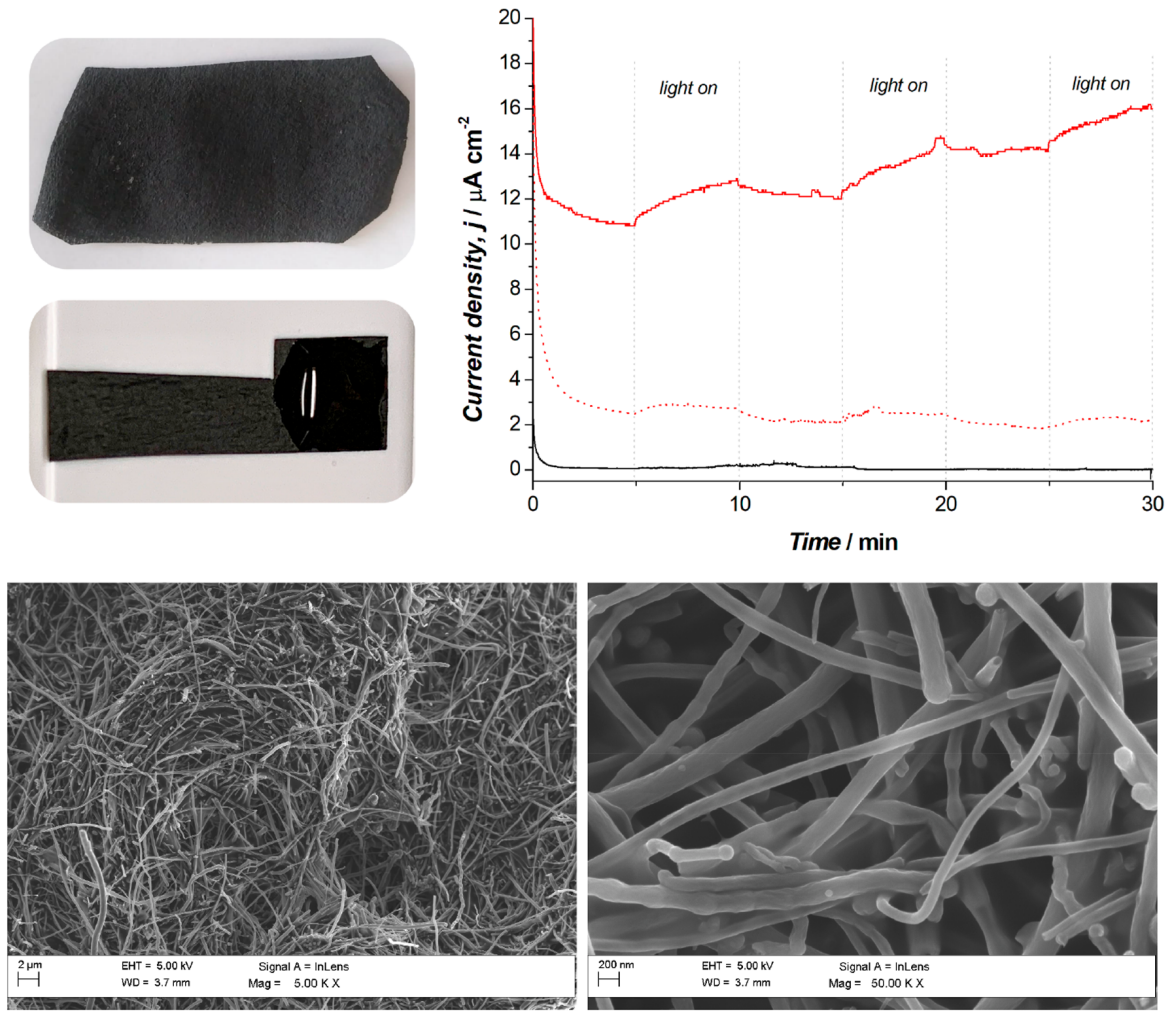
Top left: Bare PHB-based electrode and PHB-PDA-*R* biophotoanode. Top right: Amperometric *i*–*t* traces +0.32 V versus Ag|AgCl (3 M NaCl) under light/dark conditions for the biohybrid PHB-PDA-*R* photoanodes (red continuous lines). Control sterile PHB-PDA (red dashed line), and bare PHB-based electrodes (black continuous line). Bottom: SEM images of the bare PHB-based electrodes.

With the aim to validate the presented approach for redox-adhesive biohybrid photoelectrodes preparation, we further investigated its applicability with biocompatible, flexible, and cost-effective electrodes. Accordingly, the PDA-*R* biohybrid matrix was prepared on electrochemically active surfaces based on nanocarbon composites with natural synthetic biopolymer matrices. PHAs are intracellular polyesters that are accumulated by a number of microorganisms as energy and carbon reservoir, and can be produced from agri-food byproducts and waste sources in a circular biobased economy approach. Because of their natural origin, they are highly biodegradable in a wide range of conditions and highly biocompatible. Along with their environmental friendliness, PHAs are thermoplastic resins with good mechanical properties, thus being ideal candidates as structural components for composite 2D or 3D objects. The use of such biopolymers as binders for electrically conductive nanostructured carbon materials can lead to unique benefits in fabricating all-carbon biocompatible electrodes for bioelectrochemical systems. Only few studies are available on the use of PHAs as functional and structural materials for nanocarbon composite bioanodes in bioelectrochemical systems, showing promising features of these systems for the development of free-standing biocompatible all-carbon electrodes.^57^ The homogeneous, flexible, free-standing film produced by pouring a solution with a mass loading of 25 mg cm^–2^ on paper towel, and following removing the layer of paper, obtaining the electrode cut into the desired shape is shown in Figure 6 (top, left). Fifty microliters of the solution of PDA-*R* was casted on the substrate and let to dry before the electrochemical polymerization step was performed as previously discussed. Amperometric *i*–*t* traces of the PHB-PDA-*R* biophotoanode are reported in Figure 6 (top right, red continuous line), with a biophotocurrent of 1.3 ± 0.1 μA cm^–2^. Control experiments with abiotic PHB-PDA and PHB-based electrodes only were also performed (red dashed line and black line, respectively), revealing limited (0.22 ± 0.03 μA cm^–2^) and no photoresponse, respectively. The obtained results not only confirmed the applicability of the developed biohybrid matrix on different electrode substrates but also allowed a significant increase in biophotocurrent possibly due to the porous surface of the PHB-electrodes (Figure 6, bottom) that results in a higher surface area compared to glassy carbon electrodes. Interestingly, the PHB-PDA-*R* electrodes also showed a remarkably higher background current compared to the two control electrodes due to the current resulting from the heterotrophic metabolism of *R. capsulatus* for malic acid oxidation that prevails in the absence of illumination. Dark currents have been previously reported for *R. capsulatus* bioelectrodes^27,30,58^ and represent an important feature of these biohybrid electrodes enabling current generation in the absence of light. Additionally, the dark current showed a drift over time, after the first and second light cycles, toward higher values. Such drift might be due to an increased wetting of the PDA-*R* matrix deposited on the PHB-based electrode. It should be noted that the PHB-PDA-*R* system has not been optimized for maximum bio(photo)electrocatalytic performance, and future studies should focus on characterizing the detailed electrode material effects on the PDA-*R* biohybrid system. Finally, it should be underlined that the current densities achieved with the PHB-PDA-*R* biophotoanode match the highest current densities reported in literature for purple bacteria-based photoanodes employing Os-based^29^ or quinone-based^30^ artificial redox polymers while avoiding the time-consuming and separate synthesis of those polymeric matrices.

## Conclusions

A bioinspired redox-adhesive polymeric matrix obtained by one-pot dopamine polymerization with purple bacterial cells provided a sustainable and cost-effective approach for the preparation of biophotoelectrodes. The presented biohybrid system enhances photocurrent production thanks to the facilitated photoinduced electron transfer at the biotic/abiotic interface, without requiring the addition of diffusible redox mediators in the matrix, thus simplifying the system and avoiding the risk of mediator release in the environment. This could be achieved thanks to the redox-active catechol groups in PDA and the possible accumulation of endogenous quinones at the biotic/abiotic interface. The biocompatibility of the presented approach does not require the separate synthesis of a redox polymeric matrix, a significant advantage over other polymeric redox-matrices reported in literature for intact-bacteria redox mediation. This sustainable biophotoanode paves the way for the future implementation of biohybrid electrochemical systems for the sun-powered electrosynthesis of valuable chemicals as well as biosensing and bioremediation of pollutants in water environments.

## Supporting Information Available

Control cyclic voltammetries, FT-IR/ATR spectra of modified PDA, UV–vis spectrum of spent electrolyte for PDA-HQ-*R* (PDF)
