# Supplementary material for: Bio-Inspired Redox-Adhesive Polydopamine Matrix for Intact Bacteria Biohybrid Photoanodes
Source: ACS Appl Mater Interfaces. 2022 May 31;14(23):26631–41. doi: 10.1021/acsami.2c02410 (PMC9204692; doi:10.1021/acsami.2c02410)
Supplement: Supplementary file 1 — am2c02410_si_001.pdf [file am2c02410_si_001.pdf]

SUPPORTING INFORMATION

Bio-Inspired Redox-Adhesive Polydopamine Matrix for Intact Bacteria Biohybrid Photoanodes

Gabriella Buscemi,[†,‡] *Danilo Vona,*[†] *Paolo Stufano,*[§] *Rossella Labarile,*[†,‡] *Pinalysa Cosma,*[†,‡] *Angela Agostiano,*[†,‡] *Massimo Trotta,*[‡] *Gianluca M. Farinola,*[†] *Matteo Grattieri*[†,‡] *

[†] Dipartimento di Chimica, Università degli Studi di Bari "Aldo Moro", via E. Orabona 4, Bari, 70125, Italy

[‡] IPCF-CNR Istituto per i Processi Chimico Fisici, Consiglio Nazionale delle Ricerche, via E. Orabona 4, Bari, 70125, Italy

[§] CNR-NANOTEC, Institute of Nanotechnology, Consiglio Nazionale delle Ricerche, via E. Orabona 4, Bari, 70125, Italy

Corresponding Author: * Matteo Grattieri - Email: matteo.grattieri@uniba.it

TABLE OF CONTENTS

Figure S1. Control cyclic voltammetries — Page S-2

Figure S2. FT-IR/ATR spectra of modified PDA — Page S-2

Figure S3. UV-vis spectrum of spent electrolyte for PDA-HQ-*R* — Page S-3

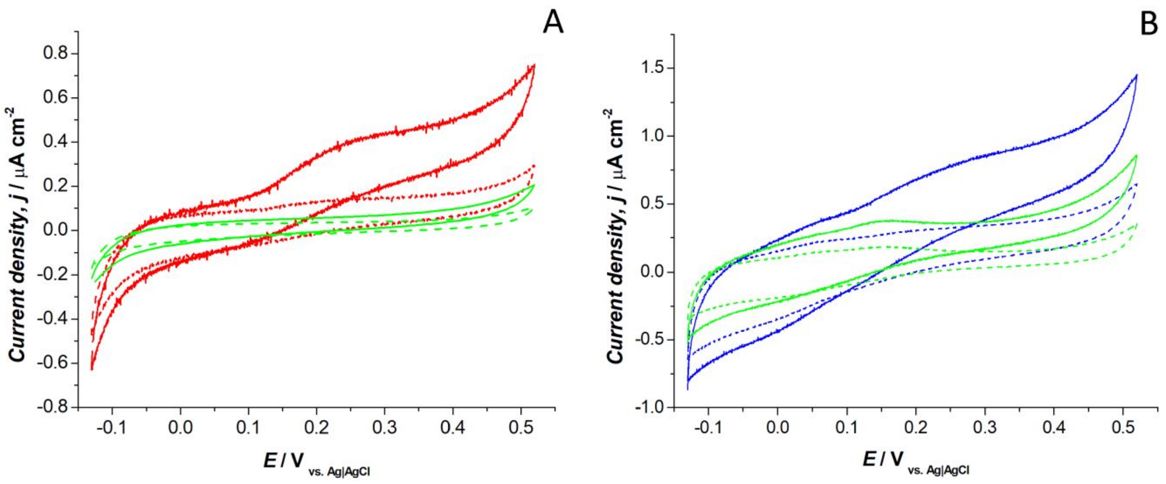

Figure S1. Cyclic voltammetries for the biohybrid photoanodes and their respective abiotic controls (green) under light (continuous lines) and dark conditions (dashed lines) for the PDA (A, red) and PDA-HQ (B, blue) systems. Scan rate: 1 mV s^{-1}; CE: Pt; RE: Ag|AgCl 3M NaCl.

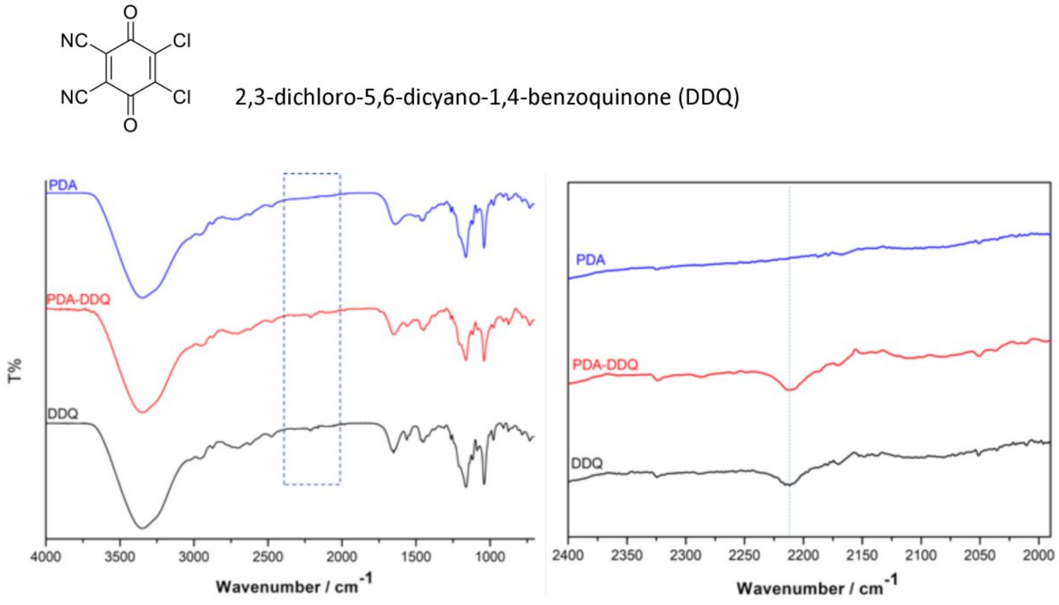

Figure S2. Left: FT-IR/ATR spectra of PDA (in MOPS buffer), PDA-2,3-dichloro-5,6-dicyano-1,4-benzoquinone (DDQ, in MOPS buffer), and DDQ only (in DMSO/MOPS). Right: magnification of the region 2400-2000 cm^{-1}.

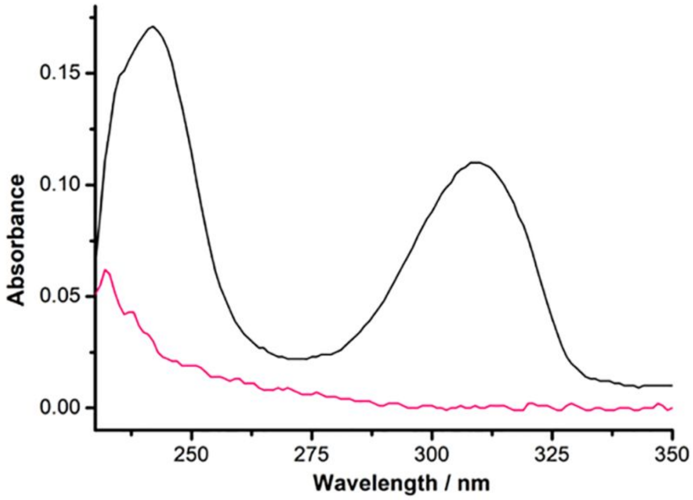

Figure S3. Uv-vis absorption spectra for 0.1 mM HQ (black) and the spent electrolyte obtained after a one hour amperometric i-t characterization of the PDA-HQ-*R* biophotoanode.
